# Time to Treatment Prediction in Chronic Lymphocytic Leukemia Based on New Transcriptional Patterns

**DOI:** 10.3389/fonc.2019.00079

**Published:** 2019-02-15

**Authors:** Adrián Mosquera Orgueira, Beatriz Antelo Rodríguez, Natalia Alonso Vence, Ángeles Bendaña López, José Ángel Díaz Arias, Nicolás Díaz Varela, Marta Sonia González Pérez, Manuel Mateo Pérez Encinas, José Luis Bello López

**Affiliations:** ^1^Health Research Institute of Santiago de Compostela (IDIS), Santiago de Compostela, Spain; ^2^Division of Hematology, Complexo Hospitalario Universitario de Santiago de Compostela, SERGAS, Santiago de Compostela, Spain; ^3^Department of Medicine, University of Santiago de Compostela, Santiago de Compostela, Spain

**Keywords:** chronic lymphocytic leukemia, time to treatment prediction, gene expression, RNAseq, machine learning, prognostic factors, *IGHV*

## Abstract

Chronic lymphocytic leukemia (CLL) is the most frequent lymphoproliferative syndrome in western countries. CLL evolution is frequently indolent, and treatment is mostly reserved for those patients with signs or symptoms of disease progression. In this work, we used RNA sequencing data from the International Cancer Genome Consortium CLL cohort to determine new gene expression patterns that correlate with clinical evolution.We determined that a 290-gene expression signature, in addition to immunoglobulin heavy chain variable region (*IGHV*) mutation status, stratifies patients into four groups with notably different time to first treatment. This finding was confirmed in an independent cohort. Similarly, we present a machine learning algorithm that predicts the need for treatment within the first 5 years following diagnosis using expression data from 2,198 genes. This predictor achieved 90% precision and 89% accuracy when classifying independent CLL cases. Our findings indicate that CLL progression risk largely correlates with particular transcriptomic patterns and paves the way for the identification of high-risk patients who might benefit from prompt therapy following diagnosis.

## Introduction

Chronic lymphocytic leukemia (CLL) is a low-grade B-cell lymphoproliferative disease with an estimated yearly incidence in western countries of about 6.9 cases per 100,000 people (1) and remarkable variation between races. The incidence of CLL is higher in men than in women and it increases progressively from the age of 35 until the last decades of life (2). Currently, CLL treatment is delayed until disease progression (bone marrow failure, organomegaly, general symptoms, or high-grade lymphoma transformation) and in the case of refractory autoimmune phenomena (3, 4). Nevertheless, with the advent of new targeted treatments such as ibrutinib (5), idelalisib (6), and venetoclax (7), it is tempting to speculate that some individuals could benefit from early intervention immediately following diagnosis, when the tumoral mass is smaller and patients have a better physical condition. Thus, improved risk stratification for patients with CLL is needed.

Recent advances in CLL genomics have discovered new drivers of disease, many of which are associated with a different clinical evolution. Deletions (6p21, 6q15, 11q, 14q24, 15q15, 17p, 18p, and 20p; gains in 2p16, 5q24, and 8q24), trisomy 12, and gene mutations (*TP53, ATM, NOTCH1, SF3B1, BIRC3, BRAF, POT1, ZNF292, NFKB2, MGA, IRF4, DDX3X, ZMYM3*, and *FUBP1*)have been repeatedly observed in the CLL genome and are linked to rapid disease progression (8) Nevertheless, immunoglobulin heavy chain variable region (*IGHV*) mutation status, which is an indirect measure of the tumor lymphocytes' maturation stage (9), is among the most important single predictive factor known to date (10). *IGHV* unmutated patients show remarkably worse prognosis than *IGHV* mutated patients (10, 11) and only a few other genomic factors have proven to be associated with clinical evolution independent of this variable. Lymphocyte maturation is such an important indicator that DNA methylation status has been used to classify CLL into three different groups that resemble different B cell maturation stages (naive B cell, intermediate, and memory B cell). This classification was shown to outperform *IGHV* status at predicting time to first treatment (TTT) (12).

Mutations, genomic aberrations, and DNA methylation patterns induce transcriptomic changes that can be measured using RNA sequencing (RNAseq), a technique that offers an opportunity to identify new biomarkers for disease progression and drug response prediction (13–15). In fact, previous efforts to improve CLL risk stratification based on RNAseq data have demonstrated impressive results (16), but the clinical application is difficult due to the expense of extensive technical and bioinformatics efforts. Therefore, there is a need for smaller transcriptomics patterns correlated with disease evolution for medical use.

In this study, we performed machine-learning based Gaussian mixture model clustering on a subgroup of genes significantly associated with TTT in order to identify transcriptional clusters with clinical implications. We studied TTT due to the lack of treatment uniformity in the International Cancer Genome Consortium (ICGC) CLL cohort and because it is a variable associated with overall survival (17). We tested our results on a 196 patient cohort and validated its clinical significance in an independent 79 patient cohort. The overall results delineated two *IGHV*-independent transcriptional clusters that stratify patients according to their risk of treatment initiation. Furthermore, we demonstrated that machine learning algorithms using gene expression data can predict patient need for treatment in the first 5 years following diagnosis. We anticipate that our findings will improve the identification of high-risk CLL patients following diagnosis.

## Materials and Methods

### Data Sources and Patient Characteristics

We applied for access to the ICGC's CLL sequencing data (18) deposited in the European Genome-Phenome Database (EGA) (19). The Data Access Committee approved access to this data under DACO-1040945. Two CLL RNA-seq cohorts were uploaded in two stages with the following accession codes: *EGAD00001001443* and *EGAD00001000258*.

The first cohort (*EGAD00001001443*, hereafter study cohort) contains RNAseq data and from CLL-purified cells of 196 individuals along with clinical data. The cohort was composed of 169 CLL, 22 monoclonal B cell lymphocytosis (MBL), and five small lymphocytic lymphoma (SLL) samples. There were 132 *IGHV* mutated cases and 64 *IGHV* unmutated cases in 119 males and 77 females. By staging at diagnosis, there were 22 MBL cases, 151 Binet Stage A cases, 14 Binet Stage B cases, and 8 Binet C stage cases.

The second cohort (*EGAD00001000258*, hereafter validation cohort) is composed of RNAseq data of CLL-purified cells from 98 individuals, of which 79 (55 males and 24 females) have publicly available phenotypic information. In this cohort there were 72 CLL, 4 SLL, and 3 MBL samples. 45 of the patients had mutated *IGHV* and 34 had unmutated *IGHV*. By staging at diagnosis, there were 3 MBL, 72 Binet Stage A, 3 Binet Stage B, and 1 Binet Stage C cases.

A summary of the patient characteristics of both cohorts can be consulted in Table 1.

**Table 1:** Patient characteristics for the test and validation cohorts.

**Category**	**Test cohort**	**Validation cohort**
Cases	196	79
Age at diagnosis (median)	63	62
Sex (% males)	60.70%	69.62
MBL	11.20%	3.79%
Binet A	77.44%	91.13%
Binet B	7.18%	3.79%
Binet C	4.10%	1.26%
IGHV unmutated	32.65%	43%
SLL	2.55%	2.04%
Proportion of progressions in the first 5 years since diagnosis	31.12%	31.64%

### Data Preprocessing and Alignment

RNAseq paired-end data were obtained from Illumina paired-end sequencing performed by the ICGC CLL consortium as described by Ferreira et al. (16) Illumina adapters were removed using *cutadapt* (20) and alignment to the human reference genome (GRCh37) was performed using *Hisat2* (21) with default specifications. We used the *Hisat2*-provided Hierarchical Graph FM index for GRCh37 with SNP and Ensembl transcript information. Bam files were sorted and indexed using *samtools* (22).

### Gene Expression Estimation

RNAseq bam files were processed in *R* (23) according to the RNAseq gene expression protocol developed by Love et al. (24) Briefly, bam files were read using *Rsamtools*, (25) followed by gene-level expression estimation using the *SummarizeOverlaps* function from the *GenomicAlignments* package. (26) Gene models in GTF format were downloaded from Ensembl (GRCh37.75 version) (27). Genes with a median read count below one were discarded.

### Statistical Analysis

We analyzed gene expression association with CLL's TTT using cox regression implemented in the *survival* package (28, 29). In this model we included the covariates donor sex and CLL stage (MBL, Binet Stage A, Binet Stage B, and Binet Stage C). Time to Treatment was calculated as the period between CLL diagnosis and the initiation of the first treatment for CLL. The day of last follow-up was used for right censoring the data of patients with incomplete follow-up.

Clustering was performed using the *Mclust* package (30) with default parameters. Briefly, *Mclust* infers the likeliest data clusters based on Gaussian Mixture Modeling fitted by an Expectation-Maximization (GMM-EM) algorithm.

Those genes with significant association with TTT in the study cohort (cox regression false discovery rate [FDR] below 5%) were selected as our initial list of genes. Variable selection was performed by adding one new gene in *p*-value ascending order to the model (starting with the first two most significant genes until reaching the top at 2,198 genes [FDR < 5%]) and computing the most likely clusters. For the sake of simplicity, we discarded the 25% least variable genes, the 50% least expressed genes and those with a high (>0.9) Spearman's rank correlation with any other gene in the input data. In the case of a highly correlated pair of genes, the one with the lowest *p*-value was discarded. In each iteration we forced Mclust to calculate the two most likely groups of samples in our data, and to select the best model according to the maximal Bayesian Information Criterion (BIC). Association with TTT calculated using cox regression (*survival* package), including *IGHV* mutation status as covariate in each iteration. *P*-value adjustment was performed with the Bonferroni method.

### Machine Learning Ensembl Construction

For *IGHV* status and need of treatment at 5 years prediction we ran boosted trees analysis using BigML applications (31) with a 2,000 tree node threshold. We chose 5 years due to the following reasons: (1) it is important to differ which patients will have progression in the first years since diagnosis; and (2) the number of cases progressing in earlier years was too small in order to train a good classificator. Varying percentages of learning rates were tested. The best model was selected based on receiver operating characteristic (ROC) curves, Precision-Recall curves, and Kolmogorov-Smirnov statistics.

## Results

### Genes Associated With Time to Treatment and Clusterization

A cox regression model was constructed with gene expression, donor sex and CLL stage at diagnosis as independent variables. 2,198 genes were found to be significantly associated with TTT (FDR < 5%) in the study cohort.

**Figure 1:**
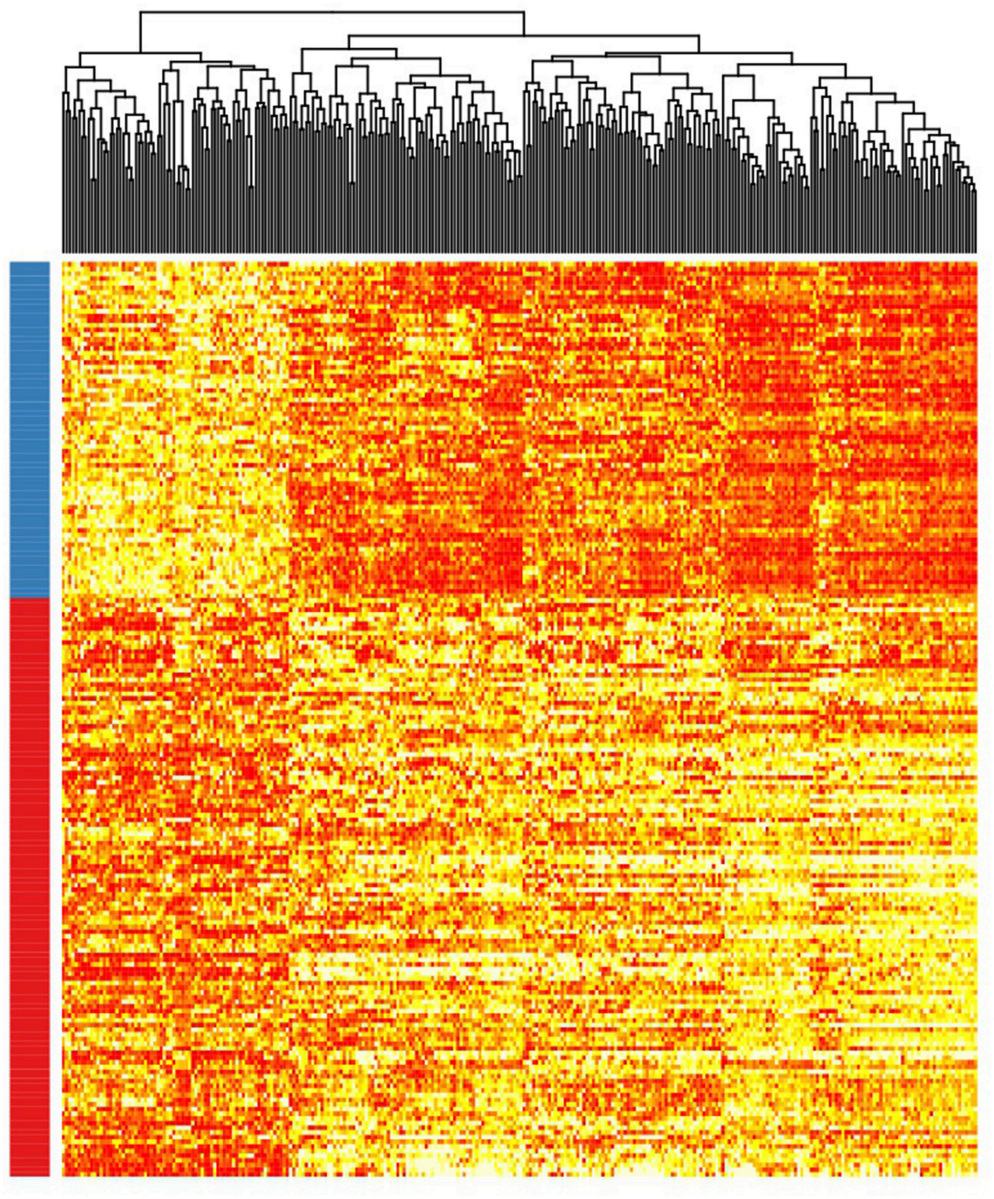
Heatmap showing the rank-transformed distribution of expression values for the 290 genes in the study cohort. Red-labeled samples on the left bar petain to C1 and blue-labeled samples pertain to C2.

**Figure 2:**
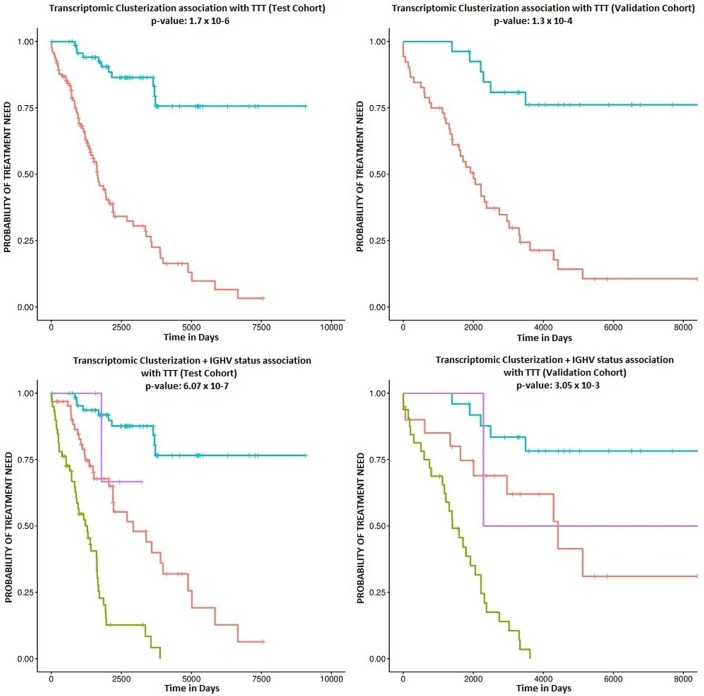
Kaplan-Meier survival plots. The upper plots show the association of C1 (red curve) and C2 (blue curve) with TTT in the study **(left)** and validation cohorts **(right)**. Corresponding *p*-values are 1.7 × 10^−6^ and 1.3 × 10^−4^. The lower plots show the association with TTT stratified by *IGHV* mutation status in the study **(left)** and validation cohorts **(right)**. The blue line indicates C2 samples with mutated *IGHV*, the purple line indicates C2 samples with unmutated *IGHV*, the red line indicates C1 samples with mutated *IGHV*, and the green line refers to C1 samples with unmutated *IGHV*.

**Figure 3:**
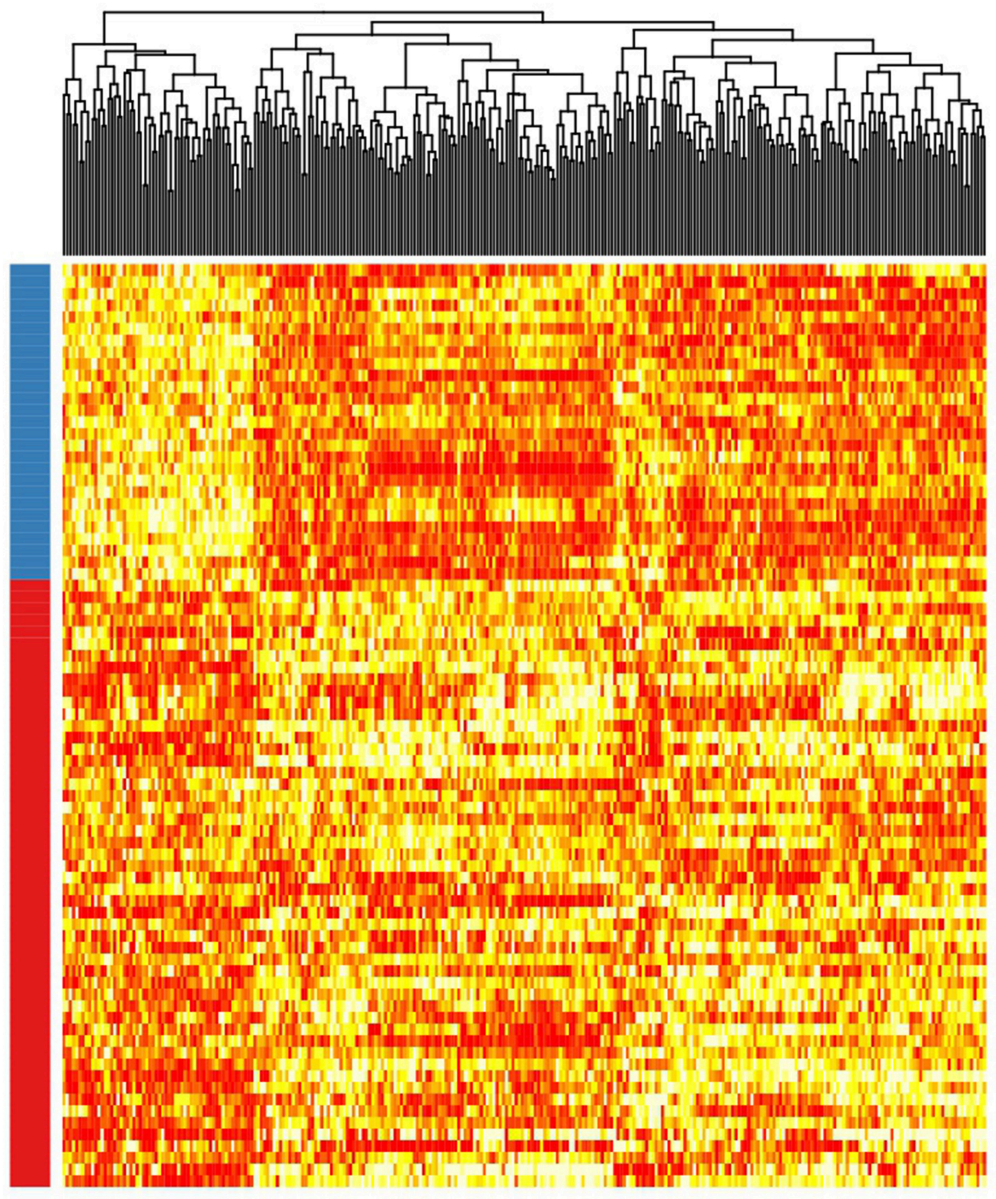
Heatmap showing the rank-transformed distribution of expression values for the 290 genes in the validation cohort. Red-labeled samples on the left bar petain to C1 and blue-labeled samples pertain to C2.

Patient clusterization based on gene expression data using a GMM-EM algorithm retrieved 19 sets of genes that clustered samples into two groups with significant associations with TTT when adjusted for *IGHV* status (Bonferroni-adjusted *p*-value < 0.01) (Supplemental Table 1). The most significant cluster (cluster 2) contained 290 transcripts (Figure 1, Supplemental Table 2) and achieved an association *p*-value of 6.4 × 10^−7^ (Bonferroni *p*-value 1.4 × 10^−3^) with the TTT variable adjusted for *IGHV* mutation status (Figure 2). A significant association was confirmed in the validation cohort (*IGHV* adjusted *p*-value 3.05 × 10^−3^) (Figures 2, 3).

According to the selected classificator, patients in cluster two (C2) had a more favorable prognosis than patients in cluster one (C1) (Hazard Ratios (HR) of−1.70 and−1.41 in the test and validation cohorts, respectively), independently of *IGHV* mutation status. Among the study cohort, roughly 36.7% of patients belonged to C2, while 34.1% of patients in the validation cohort clustered within C2. C2 involved 51.5% of *IGHV*-mutated patients and 6.4% of *IGHV*-unmutated patients in the study cohort, as well as 55.5% of *IGHV*-mutated patients and 5.8% of *IGHV*-unmutated patients in the validation cohort.

### Machine Learning for Treatment Free Survival Prediction

We were interested in a machine learning (ML) classifier that could predict which patients would require CLL therapy in the first years following diagnosis. We constructed model ensembles with all genes associated with TTT in Cox regression at a FDR of 5%. We also tested different learning rates (0.5, 1, 2.5, 5, and 10%). 222 patients had a follow-up period >5years or had been treated in the first 5 years following diagnosis, and we divided them into a training set (80% of patients, composed of 146 patients from the study cohort and 31 patients from the validation cohort) and a test set (20% of patients, composed of 45 patients from the validation cohort). ROC AUC and Precision-Recall AUC plots were evaluated to select the best results.

**Figure 4:**
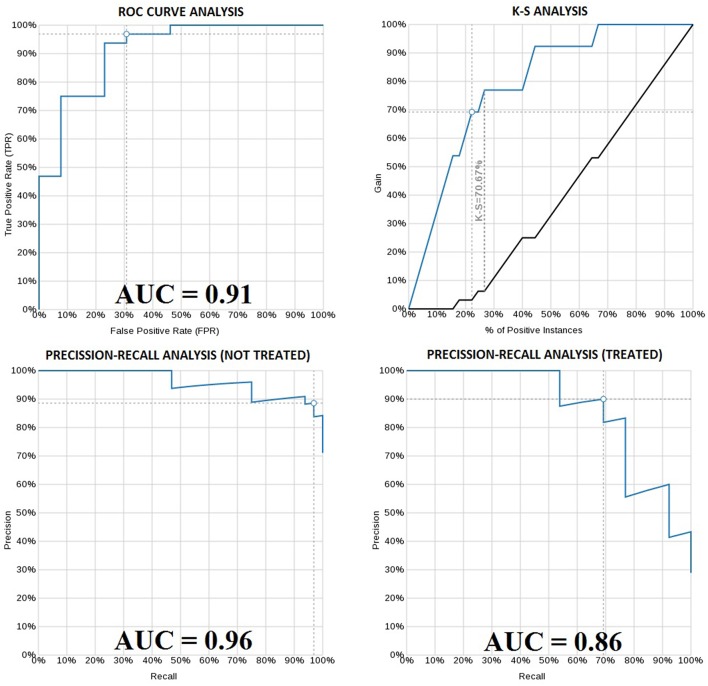
ROC curve of the boosted-tree Ensembl model for 5 year treatment need prediction **(upper left)**. Kolmogorov-Smirnov plot for the same model **(upper right)**. Precision-Recall plot for the 5 year not-treated **(lower left)** and treated **(lower right)** patients according to the same model. White dots in each graph indicate the probability threshold (in this case 50%), which is the point reflecting the best classification accuracy of the patients.

The best model used a 2.5% learning rate and 2,000 tree nodes. It achieved 90% precision at identifying patients that needed treatment in 5 years with 69.23% recall, and 88.57% precision at identifying those patients that did not require treatment in 5 years with 96.88% recall. We only detected 1 false positive case (3.1% False Positive Rate) and 4 false negatives (30% False Negative Rate). Average precision was 89.29%, accuracy was 88.89% and ROC area under the curve (AUC) was 0.911 (Figure 4 and Table 2). Precision-Recall AUC was 0.860 and 0.959 for predicting which patients would or would not need treatment within this period, respectively. In each case, the results mostly overlapped with the area under the convex hull (AUCH).

**Table 2:** Confusion Matrix for the boosted-tree Ensembl model predicting the 5 year need of treatment.

**5 year treat. need**	**Not needs treat**.	**Needs treat**.	**Actual**	**Recall(%)**
Not needs treat.	31	1	32	96.88
needs treat.	4	9	13	69.23
predicted	35	10	45	83.05^**^
precision	88.57%	90.00%	89.29%^*^	88.89^***^

* *Average precision*.

** *Average recall*.

*** *Accuracy probability threshold = 50%*.

## Discussion

The main aim of this study was to identify new transcriptomic patterns in order to improve CLL patient risk stratification. We used the GMM-EM algorithm to stratify patients in two clusters with remarkably different clinical behavior based on the expression of 290 genes, and we observed that this pattern was independent of *IGHV* mutation status. Interestingly, we identified a group of CLL patients with mutated *IGHV* and a low-risk transcriptomic profile that only need treatment in approximately 25% of the cases during disease evolution. Two additional groups (one composed of patients with mutated *IGHV* and a high-risk transcriptomic profile and the second composed of unmutated *IGHV* patients with a low-risk transcriptomic profile) have similar intermediate evolution, while a final group (composed of patients with unmutated *IGHV* and an adverse transcriptomic profile) has the highest probability of treatment need in the first years following diagnosis. These results are concordant with previous reports in the field. For example, Yepes et al. (32) reported a division of CLL cases in two groups based on microarray transcriptome characterization through unsupervised clustering analysis, which was validated in 4 independent cohorts. Similarly, Friedman et al. (33) described a 180 probe classifier based on microarray data that also divided two clusters of CLL patients independently of *IGHV* mutation status. Our findings are also similar to those published by Ferreira et al. (16), who described two gene expression clusters that show *IGHV* mutation-independent association with TTT using an early release of the ICGC CLL cohort. Nevertheless, there are remarkable differences between our analysis and that of Ferreira et al, Yepes et al. and Friedman et al. Firstly, our clusterization is based on a transcriptional pattern of a small subgroup of genes that facilitates its future applicability, whilst those of Ferreira et al. and Yepes et al. are based on whole transcriptome analysis. Secondly, our classifier is based on RNAseq data, a technology that has outperformed microarray analysis in most fields. With the use RNAseq it will be possible to couple transcriptome clusterization with targeted gene mutation detection, stereotyped B cell receptor expression or *IGHV* hypermutation status analysis.

We also describe a novel artificial intelligence algorithm that can predict a CLL patient's need for therapy during the first 5 years following diagnosis with high precision and accuracy. This is in line with other ML applications to oncologic malignancies that are starting to change paradigms in patient risk stratification and drug response prediction. For example, Aziz et al. (34) recently reported the identification of a ML model that integrates clinical and genomic data from patients with myelodysplastic syndrome (MDS). This model outperformed all commonly used prediction models in the field of MDS. Similarly, Yousefi et al. (35) used bayesian-optimized deep learning for survival prediction in pan-cancer analysis, showing not only better performance than other state-of-the-art methods, but also improved predictability of cancer survival through transfer learning in different types of cancer genomic data. Thus, it is likely that ML-driven algorithms applied to genomic and transcriptomic data will be used in the near future for the identification of “smoldering” CLLs that may benefit from early intervention.

RNAseq is a powerful technique that can sequence the whole transcriptome at an increasingly lower cost. Targeted RNAseq is being developed for clinical application, with the additional possibility of testing for gene mutations and fusion genes in the same technique. Therefore, defining reproducible gene expression patterns with clinical implications is a strategy that can close the gap between research and the clinical practice. Here we present patterns of gene expression that can improve CLL patient risk stratification with a relatively small set of the transcriptome. These results may pave the way for the design of new treatment strategies involving early CLL treatment in high-risk patients before disease progression.

## Author Contributions

AMO designed the study performed research. AMO, BAR, JDA, and JBL analyzed the data. AMO wrote the paper. NAV, ABL, NDV, MGP, and MPE reviewed the paper.

### Conflict of Interest Statement

The authors declare that the research was conducted in the absence of any commercial or financial relationships that could be construed as a potential conflict of interest.

## Supplementary Material

Supplemental Table 1 This table represents the original 19 clusters with Bonferroni *p*-values < 0.01.

Supplemental Table 2 List of the 290 genes used for C1 vs. C2 clusterization.
